# Diet-Induced Obesity in Mice Overexpressing Neuropeptide Y in Noradrenergic Neurons

**DOI:** 10.1155/2012/452524

**Published:** 2012-10-18

**Authors:** Suvi T. Ruohonen, Laura H. Vähätalo, Eriika Savontaus

**Affiliations:** ^1^Department of Pharmacology, Drug Development, and Therapeutics and Turku Center for Disease Modeling, University of Turku, Itäinen Pitkäkatu 4B, 20520 Turku, Finland; ^2^Fin Pharma Doctorate Program Drug Discovery Section, University of Turku, Itäinen Pitkäkatu 4B, 20520 Turku, Finland; ^3^Unit of Clinical Pharmacology, Turku University Hospital, Itäinen Pitkäkatu 4B, 20520 Turku, Finland

## Abstract

Neuropeptide Y (NPY) is a neurotransmitter associated with feeding and obesity. We have constructed an NPY transgenic mouse model (OE-NPY^DBH^ mouse), where targeted overexpression leads to increased levels of NPY in noradrenergic and adrenergic neurons. We previously showed that these mice become obese on a normal chow. Now we aimed to study the effect of a Western-type diet in OE-NPY^DBH^ and wildtype (WT) mice, and to compare the genotype differences in the development of obesity, insulin resistance, and diabetes. Weight gain, glucose, and insulin tolerance tests, fasted plasma insulin, and cholesterol levels were assayed. We found that female OE-NPY^DBH^ mice gained significantly more weight without hyperphagia or decreased activity, and showed larger white and brown fat depots with no difference in UCP-1 levels. They also displayed impaired glucose tolerance and decreased insulin sensitivity. OE-NPY^DBH^ and WT males gained weight robustly, but no difference in the degree of adiposity was observed. However, 40% of OE-NPY^DBH^ but none of the WT males developed hyperglycaemia while on the diet. The present study shows that female OE-NPY^DBH^ mice were not protected from the obesogenic effect of the diet suggesting that increased NPY release may predispose females to a greater risk of weight gain under high caloric conditions.

## 1. Introduction

Neuropeptide Y (NPY) is one of the most common peptides in the brain and an abundant neurotransmitter in the peripheral sympathetic nervous system (SNS). NPY has been linked to several disorders associated with metabolic syndrome. It plays a well-established role in the hypothalamic control of body energy balance by promoting feeding and lipid storage in white adipose tissue (WAT) [1]. However, in pair-fed rats, central NPY administration still leads to increased fat accumulation, which suggests that NPY has an important role in promoting adiposity independent of food intake [2]. NPY outside the hypothalamus in the regulation of energy homeostasis has not been widely studied, although NPY and its receptors are located in key peripheral tissues, such as WAT, liver, and pancreas. NPY inhibits lipolysis in adipocytes via Y_1_ receptors [3], and could modulate adipose tissue expansion by regulating angiogenesis [4]. NPY also inhibits insulin release via pancreatic Y_1_ receptors [5]. On the other hand, NPY in the brainstem could modulate sympathetic tone, which is known to have multiple effects on energy homeostasis. To address the role of NPY colocalized with noradrenaline in SNS and brain noradrenergic neurons, we created a transgenic mouse model (OE-NPY^DBH^ mouse), where NPY is overexpressed under the dopamine-betahydroxylase (DBH) promoter. This resulted in moderately increased levels of NPY protein (1.3–1.8-fold) peripherally in adrenal glands and centrally in noradrenergic neurons of the brainstem, but not in the hypothalamus [6]. The OE-NPY^DBH^ mice displayed increased adiposity and liver triglyceride accumulation without changes in body weight, food consumption, or physical activity on a normal chow diet [6]. Hyperinsulinemia and impaired glucose tolerance develop with age and increased adiposity in these mice [6]. The increased adiposity was observed in both genders at all ages studied, but the major metabolic complications such as impaired glucose tolerance, hyperinsulinemia, and hepatosteatosis were only present in male OE-NPY^DBH^ mice. Furthermore, the OE-NPY^DBH^ mice showed elevated sympathetic tone and increased responses to stress [7], and increased susceptibility to arterial thickening after vascular injury [8]. Others have shown that stress-induced activation of the NPY system combined with a high-calorie diet results in augmented obesity [4]. The primary finding of Kuo et al. was increased fat mass without change in food intake or body weight after two weeks of daily stress and high fat diet. Prolonged stress and diet led to liver steatosis, impaired glucose tolerance and obesity, which were attenuated by local Y_2_R antagonist administration and fat-targeted *Y*_2_-gene knockdown procedure. These data combined show that extrahypothalamic NPY regulates adiposity independent of food intake and that NPY together with stress are potent factors in several pathways leading to a complex disorder called the metabolic syndrome.

In humans, association studies of a polymorphism in the *NPY* gene have linked NPY to metabolic disturbances. The *NPY*1228T > C polymorphism (rs16139), which causes an amino acid substitution of Leucine 7 to Proline 7 (L7P) in the signal peptide [9] is functional, enhancing the secretion of NPY [10, 11]. It is associated with an earlier onset of type 2 diabetes in carriers of L7P [12], as well as with other traits of the metabolic syndrome [13] especially in obese subjects [14]. Recently, it was reported that there is a gender difference in the NPY-mediated effects of the Proline 7 allele [15]. The associations of the L7P seem to be more pronounced in men than women, which is in agreement with our findings in OE-NPY^DBH^ mice indicating that males develop more severe metabolic changes.

Human obesity and metabolic syndrome are often due to lifestyle changes including increased sedentary work and decreased physical activity, and increased accessibility and affordability of dense, high-calorie foods. An increase in dietary fat and sucrose content has been shown to produce hyperglycaemia and obesity in various strains of mice and rats. In the present study, we exposed male and female OE-NPY^DBH^ and wildtype (WT) control mice to an energy-dense Western-type diet for seven weeks, and compared the genotype and sex differences in the impacts of the diet on the development of obesity, insulin resistance, and impaired glucose tolerance. Based on the findings in these mice, the possible reasons, that is, food intake and physical activity, were studied further in the OE-NPY^DBH^ female mice.

## 2. Materials and Methods

### 2.1. Experiment 1

Eight-week-old male and female OE-NPY^DBH^ mice heterozygous for the NPY transgene [6] and their WT littermates on a C57Bl/6N genetic background were used. Animal care was in accordance with the guidelines of the European Convention for the Protection of Vertebrate Animals used for Experimental and other Scientific purposes (Council of Europe no. 123, Strasbourg 1985), and all experimental procedures were approved by the institutional animal care and use committee. OE-NPY^DBH^ mice are stress-sensitive [7], and therefore the mice in this study were housed with their same-sex siblings instead of single cages and maintained on a 12-h light/dark cycle (lights on at 6 am) with free access to food and water. The number of animals in the male groups was 9-10, and in the female groups 15-16. The mice were placed on a Western-type diet (42% kcal fat from milk fat, 43% carbohydrates from sucrose and corn starch, 15% protein, and 0.15% supplementary cholesterol, code 829100, Special Diets Services, Essex, UK) and fed *ad libitum* for seven weeks. The weight gain was monitored weekly. 

After five weeks on the diet, the mice were fasted from 6:00 h to 10:00 h, and administered intraperitoneally (ip) with glucose (10% w/v, 1 g kg^−1^ body weight) for glucose tolerance test (GTT). Tail vein blood glucose was measured immediately before the injection and at 20, 40, 60, and 90 min with a glucose analyzer (Precision Xtra, Abbott Diabetes Care, Abbott Park, IL, USA). Areas under the resultant curves (AUC) were calculated with the trapezoidal method in GraphPad Prism 5.01 software. The induction of hyperglycemia was defined as a blood glucose level over 13.8 mmol L^−1^ (250 mg dL^−1^) in mice following a fast started on the morning of the experiment as guided by mouse metabolic phenotyping centers (http://www.mmpc.org/) established by the National Institutions of Health (NIH) and by the Animal Models of Diabetic Complications Consortium (AMDCC, http://www.amdcc.org/). The same level of fasted blood glucose in determination of hyperglycaemia in mice has been used by others as well [16, 17]. After six weeks on the diet, the mice were fasted for 1 h. Recombinant human insulin (Protaphane, Novo Nordisk) at a dose of 0.5 or 1.0 IU kg^−1^ was injected ip for insulin tolerance test (ITT). Blood glucose was measured immediately before the injection and at 20, 40, and 60 min after the injection. 

At sacrifice, the mice were fasted from 6:00 h to 10:00 h, the rectal temperature was measured (Ellab, Roedovre, Denmark), and the animals were anesthetized with ketamine (Ketalar 75 mg kg^−1^) and medetomidine (Domitor 1 mg kg^−1^). Terminal blood samples were obtained from inferior vena cava with 1 mL syringe and 23 G needle into heparinized tubes. Plasma was centrifuged (4000 rpm, 10 min) and stored at −70°C until analyzed. WAT weight was determined by collecting the subcutaneous, epididymal/gonadal, and retroperitoneal fat pads. The amount of fat was calculated as a sum of these different WAT depots. In addition, brown fat and liver weights were determined, and the tissues were snap frozen in liquid nitrogen for further analyses. Liver triglyceride content was measured as described previously [6]. Brown fat samples were cryo-sectioned on microscopic slides and stained with hematoxylin and eosin (H&E) for standard morphology. Plasma concentrations of insulin (Mercodia Ultrasensitive Mouse Insulin ELISA, Mercodia AB, Uppsala, Sweden) and total cholesterol (BioVision Cholesterol Quantitation kit, BioVision Inc., Mountain View, CA, USA) were determined according to the manufacturers' instructions.

### 2.2. Brown Adipose Tissue (BAT) Thermogenic Capacity Analysis with Real-Time Quantitative PCR (qPCR) 

Total RNA in BAT was extracted with Trizol Reagent (Invitrogen, Carlsbad, CA, USA) combined with DNase treatment (TURBO DNA-free Kit, Ambion Inc., Austin, TX, USA). RNA was converted to cDNA with High Capacity RNA-to-cDNA Kit (Applied Biosystems) according to the manufacturer's instructions. Predesigned TaqMan Gene Expression assay (Applied Biosystems, assay ID Mm01244861_m1) for uncoupling protein-1 (UCP-1) was used to analyze the mRNA levels in BAT. UCP-1 levels were quantitated relatively to the housekeeping gene *β*-actin, (Mouse ACTB, VIC/MGB Probe, Primer Limited with 7300 Real-Time PCR System Applied Biosystems). Relative C_T_ method and the formula 2^−ΔΔCT^ were used as the quantitation method.

### 2.3. Experiment 2

In order to study the genotype differences in the female gender in more detail, another set of female mice age-matched to experiment 1 were studied. This time we used OE-NPY^DBH^ mice homozygous for the transgene and C57Bl/6N WT female mice. The production of the homozygous line was done as follows. Heterozygous OE-NPY^DBH^ mice were bred together in order to produce WT, heterozygous and homozygous pups, which were genotyped by qPCR. Genomic DNA was isolated from tail biopsies with a commercial kit (Puregene DNA Purification Kit, Gentra, Minneapolis, MN, USA). Forward (5′-TGGCTGGAGTGCGATCTTC-3′) and reverse (5′-GAGTTTGACCGTCTACGTGC-3′) primers, and the TaqMan-MGB probe (6FAM-CCGATACTGTCGTCGTC-MGB) were designed for the LacZ reporter gene in the transgene construct with the Primer Express 3.0 software (Applied Biosystems). LacZ levels were quantitated relatively to the housekeeping gene *β*-actin, (Mouse ACTB, VIC/MGB Probe, Primer Limited). Homozygous OE-NPY^DBH^ and littermate WT mice were selected for breeding (OE-NPY^DBH^ × OE-NPY^DBH^ and WT × WT), thus creating purely transgenic or WT lines housed separately. The first litters from the homozygous and WT mouse lines were genotyped as described above to verify all pups had the same genotype for the transgene. 

The female mice (*n* = 7–12) were placed on the Western-type diet for five weeks and their weight gain and food consumption were measured on a weekly basis. In addition, after two weeks on the diet, a 24-h spontaneous physical activity along with food intake was monitored with a photo-beam recording system (San Diego Instruments, San Diego, CA, USA) in 10 minute intervals. Mice were placed in single cages and had a 23-h adjustment period before monitoring the 24-h activity. Whole body fat mass was measured *in vivo* after the five-week period with an EchoMRI-700 (Echo Medical Systems, Houston, TX, USA).

### 2.4. Reproduction Analyses

The reproduction history of all three genotype lines was followed and the number of total pups as well as female versus male pups was analyzed. The number of WT, heterozygous OE-NPY^DBH^, and homozygous OE-NPY^DBH^ dames used in breeding was 45, 26, and 34, respectively. 

### 2.5. Statistical Analyses

The results were analyzed with a Student's parametric *t*-test or with a Mann-Whitney's nonparametric *U*-test to compare the groups of OE-NPY^DBH^ and WT mice. Weight gain, GTT and ITT were analyzed with two-way ANOVA for repeated measurements and Bonferroni posthoc tests.  *χ* ^2^  test was used to calculate the statistical difference in the number of male mice with hyperglycaemia as 0 = fasting glucose <13.8 mmol L^−1^ or 1 = fasting glucose >13.8 mmol L^−1^. Statistical analyses were carried out using GraphPad Prism 5.01 (GraphPad Software, San Diego, CA, USA). Data are presented as means ± SEM for the indicated number of observations. Means were considered significant when *P* < 0.05.

## 3. Results

### 3.1. Experiment 1: Body Weight Gain during the Diet

**Figure 1:**
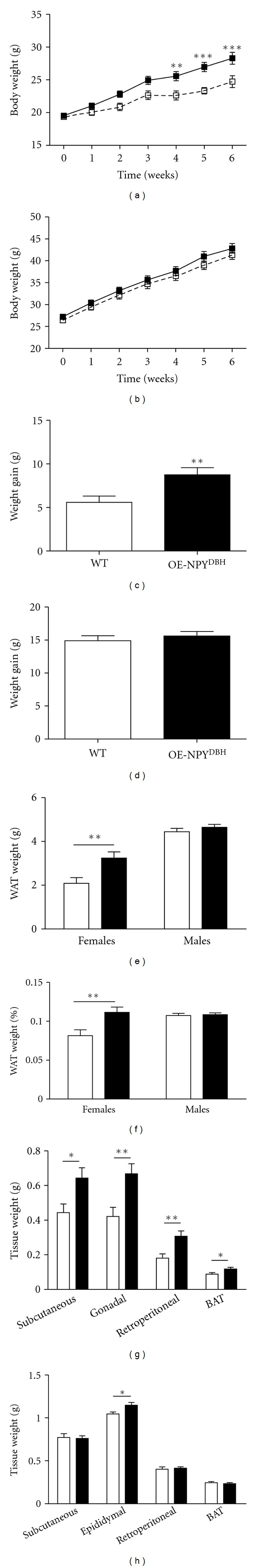
The effect of the Western-type diet on body weight gain and adiposity in wildtype (WT) and heterozygous OE-NPY^DBH^ mice. Body weight gain curves (a, b), total weight gain (c, d), and fat tissue weights (e–h) were measured as described in *Experiment *1 in Section 2, and presented here with females in the left panel (a, c, e, g) and males on the right (b, d, f, h). (a, c) Heterozygous female OE-NPY^DBH^ mice gained significantly more weight during the diet in comparison with their WT littermates. (b, d) The male mice were equally susceptible to weight gain in both genotypes. (e) Mean total WAT weights, (f) total WAT per body weight in percentages, and (g-h) different WAT subclass and BAT weights in female (e, g) and male (f, h) wildtype and OE-NPY^DBH^ mice. Values are expressed as means ± SEM. *n* = 15, WT females; *n* = 16, OE-NPY^DBH^ females; *n* = 9, WT males; *n* = 10, OE-NPY^DBH^ males. White squares and bars = WT; Black squares and bars = OE-NPY^DBH^. *P < 0.05 with a Student's *t*-test; **P < 0.01 with a Bonferroni posthoc test in repeated measures two-way ANOVA (a) or with a Student's *t*-test (c–g); ***P < 0.001 with a Bonferroni posthoc test in repeated measures two-way ANOVA.

Female OE-NPY^DBH^ mice gained more weight compared with their WT littermates on the Western diet (Figures 1(a) and 1(c)). In males, prominent weight gain was observed in both genotypes, but no difference in the weight gain pattern was observed between the OE-NPY^DBH^ and WT mice (Figures 1(b) and 1(d)). The female transgenic mice also had larger WAT and BAT mass as measured by individual fat pads or the sum of WAT pads compared with WT female mice (Figures 1(e) and 1(g)). In males, a significant difference between the genotypes was only observed in the epididymal WAT weight (Figures 1(f) and 1(h)).

### 3.2. Experiment 1: GTT

**Figure 2:**
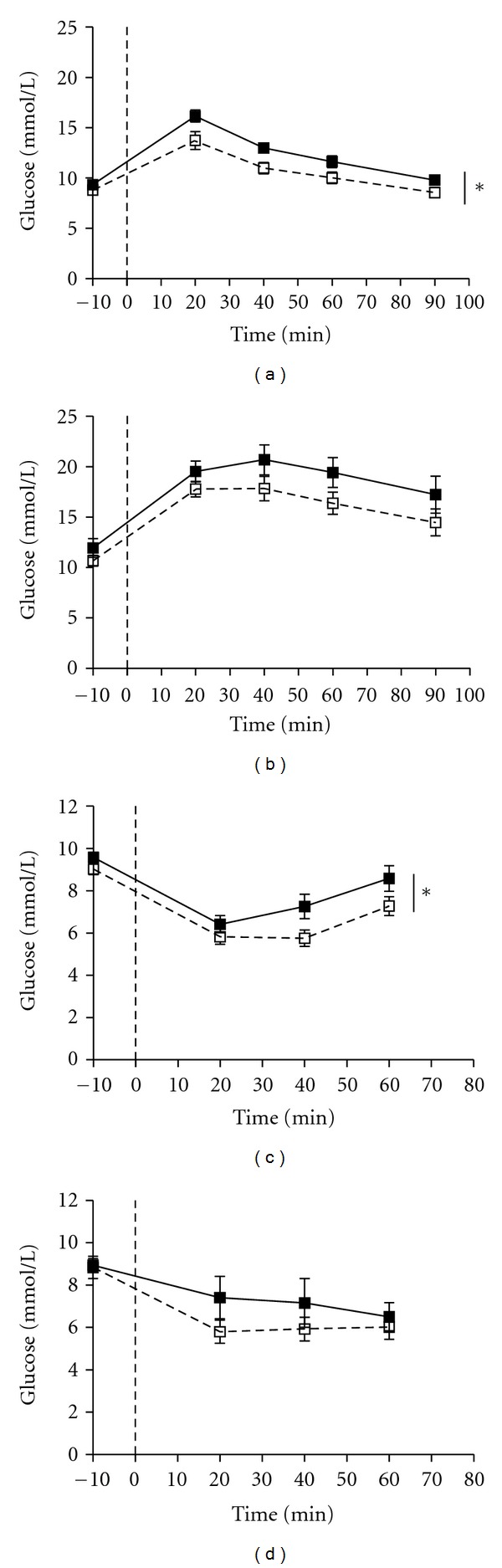
The effect of the Western-type diet on glucose homeostasis in wildtype (WT) and OE-NPY^DBH^ mice. Intraperitoneal glucose (a, b) and insulin (c, d) tolerance tests (GTT and ITT, resp.). Females are presented in the left panel (a, c) and males on the right (b, d). Mean ± SEM blood glucose values are shown at each studied time point in GTT (baseline, 20, 40, 60, and 90 min) and ITT (baseline, 20, 40, and 60 min) in female (*n* = 15, WT; *n* = 16, OE-NPY^DBH^) and male (*n* = 9, WT; *n* = 10, OE-NPY^DBH^) mice. The administration of 1.0 g kg^−1^ glucose (a, b) and insulin 0.5 IU kg^−1^ (c) or 1.0 IU kg^−1^ (d) is marked at 0 min. White squares = WT; black squares = OE-NPY^DBH^. *P < 0.05 with repeated measures two-way ANOVA.

Glucose tolerance was significantly impaired in female OE-NPY^DBH^ mice compared with WT mice, as shown by the much greater rise in blood glucose in OE-NPY^DBH^ mice over the studied 90 min time period following administration of glucose (Figure 2(a)). In addition, the AUC value in OE-NPY^DBH^ mice was significantly higher than in WT controls (Table 1). In male mice, the glucose tolerance was similar between the genotypes and thus no statistical significance between the groups was reached (Figure 2(b)). However, four out of ten OE-NPY^DBH^ male mice, but none of the nine WT controls, had a 4-h fasting glucose value over 13.8 mmol L^−1^. Thus, they were considered to show severe hyperglycaemia [16, 17]. This difference reached statistical difference of *P* < 0.05 with the  *χ* ^2^  test. There was no difference in GTT AUC values between the genotypes in males (Table 1).

### 3.3. Experiment 1: ITT

Insulin sensitivity was assessed by investigating the action of exogenous insulin on blood glucose levels. Blood glucose levels were higher in female OE-NPY^DBH^ mice compared with their WT controls at all investigated time points after intraperitoneal injection of insulin (Figure 2(c)). Insulin was first administered with 0.5 IU/kg, which was sufficient in females but had no effect on circulating glucose levels in males. Hence, the experiment was repeated with a higher dose of 1.0 IU kg^−1^. This dose decreased blood glucose on average by 35% in eight out of nine WT and seven out of ten OE-NPY^DBH^ male mice (Figure 2(d)), whereas the rest of the mice (1/9 of WT and 3/10 of OE-NPY^DBH^) were resistant to the effect of insulin, that is, the blood glucose levels were the same or even higher than at baseline. No significant difference between the genotypes in blood glucose values after insulin dosing in males was observed.

### 3.4. Experiment 1: Plasma Insulin and Cholesterol Concentrations

Circulating insulin and total cholesterol levels were determined after a 4-h fast. No difference in either parameter was observed between the genotypes in males or females (Table 1).

### 3.5. Experiment 1: Liver Triglyceride Content

Liver weights were measured at sacrifice. The female OE-NPY^DBH^ mice had significantly heavier livers *per se* (WT: 1.1 ± 0.07; OE-NPY^DBH^: 1.3 ± 0.06 grams; *P* < 0.05), but when the weights were corrected with body weights, the statistical difference was lost (data not shown). In males, the actual (WT: 2.5 ± 0.21; OE-NPY^DBH^: 2.8 ± 0.22 grams; *P* = NS) and normalized liver weights were identical between the genotypes. Hepatic lipids were determined by assessing their triglyceride contents. Gross visual examination of the livers suggested steatosis as observed by a pale abnormal color with naked eyes. No difference in triglyceride levels between the genotypes in males or females was observed (Table 1).

### 3.6. Experiment 1: Body Temperatures

Rectal body temperatures did not differ between the genotypes in males (WT: 35.4 ± 0.24; OE-NPY^DBH^: 35.7 ± 0.29 degrees centigrade) or females (WT: 35.9 ± 0.36; OE-NPY^DBH^: 35.7 ± 0.37 degrees centigrade).

### 3.7. Experiment 1: Brown Fat UCP-1 Expression Levels and Tissue Morphology

**Figure 3:**
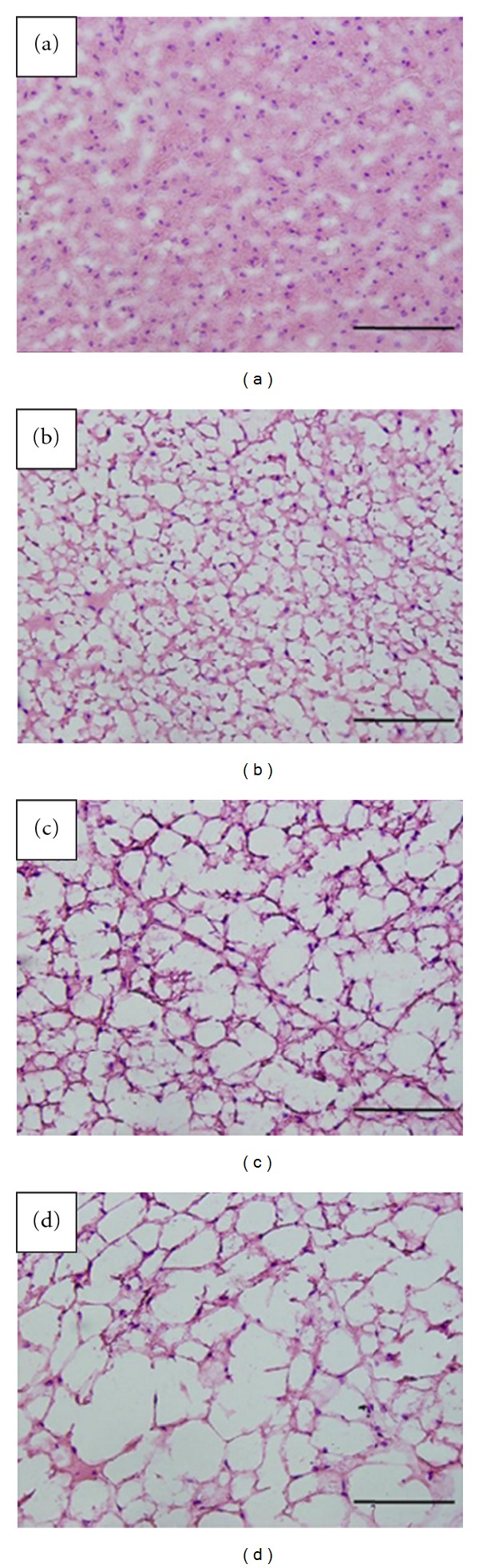
Brown adipose tissue (BAT) morphology after seven weeks on the Western-type diet. (a) WT female, (b) OE-NPY^DBH^ female, (c) WT male, and (d) OE-NPY^DBH^ male. Scale bar is 100 *μ*m.

UCP-1 expression levels in BAT were measured with qPCR by using the average mRNA expression of the WT group as a calibrator. No difference in UCP-1 levels in males (WT: 1.13 ± 0.18; OE-NPY^DBH^: 1.16 ± 0.15 fold mRNA expression; *P* = NS) or females (WT: 1.09 ± 0.13; OE-NPY^DBH^: 1.24 ± 0.16 fold mRNA expression; *P* = NS) were observed. The tissue morphology showed atypical BAT tissue with lipid deposition in both male groups and in the OE-NPY^DBH^ female group whereas the WT females showed normal BAT morphology (Figure 3).

### 3.8. Experiment 2: Body Weight Gain, Feeding Behavior and Locomotor Activity during the Diet

**Figure 4:**
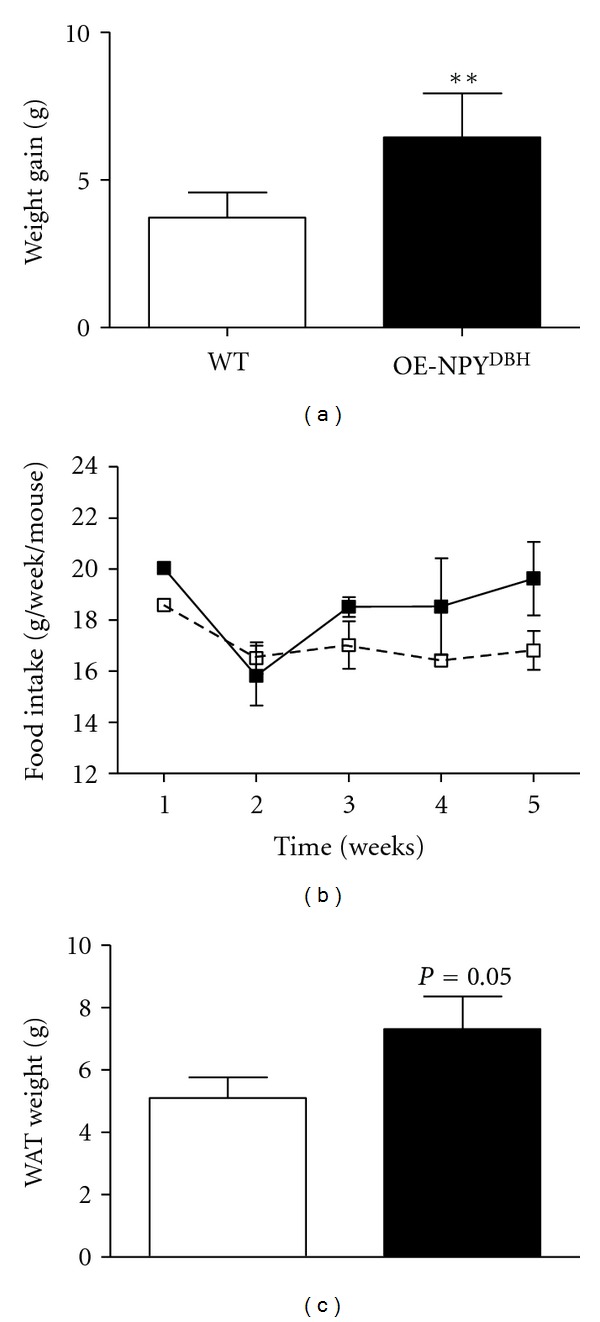
The effect of the Western-type diet on body weight gain and adiposity in wildtype (WT) and homozygous OE-NPY^DBH^ mice. Total weight gain (a), food intake (b), and total amount of fat tissue (c) were measured as deascribed in *Experiment *2 in Section 2. Values are expressed as means ± SEM. *n* = 12, WT females; *n* = 7, OE-NPY^DBH^ females. White squares and bars = WT; black squares and bars = OE-NPY^DBH^.   **P < 0.01 with a Mann-Whitney test; *P* = 0.05 with a Student's *t*-test.

**Figure 5:**
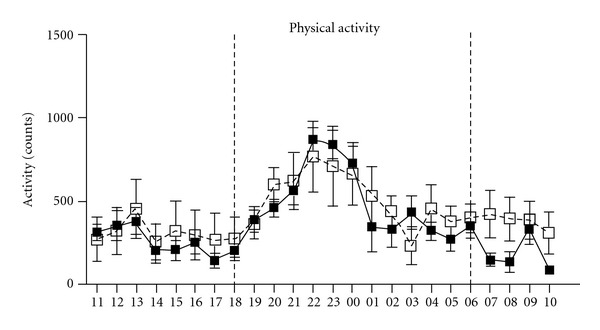
Physical activity during the Western-type diet in wildtype (WT) and homozygous OE-NPY^DBH^ female mice. The total activity was calculated as the sum of horizontal and vertical movement counts over the 24-h period starting at 11:00 o'clock a.m. The dark period (18:00–06:00 h) is marked with vertical lines. Mean ± SEM count values are shown for each hour (*n* = 12, WT; *n* = 7, OE-NPY^DBH^). White squares = WT; Black squares = OE-NPY^DBH^.

In order to verify and to find the reason for the findings in females, the early steps of the weight gain and adiposity were further studied in a homozygous OE-NPY^DBH^ line. A similar weight gain pattern was observed as in the heterozygous mice in comparison with WT controls, that is, the weights start to differ after four weeks on the diet and are significantly higher at five weeks from the beginning of the diet (Figure 4(a)). Food intake measured weekly in group-housed (food consumed divided by the number of animals) (Figure 4(b)) or in 47-h individually housed mice (WT: 6.7 ± 0.7; OE-NPY^DBH^: 6.3 ± 1.2 grams; *P* = NS) was not different between the genotypes. A whole body EchoMRI analysis was performed at week five to measure the amount of fat *in vivo*. The OE-NPY^DBH^ females showed increased adiposity compared with WT mice (Figure 4(c)), which explains the difference in body weights. 24-h Locomotor activity of female WT and OE-NPY^DBH^ mice measured in photo-beam cages after two weeks on the diet revealed no differences between the genotypes either in horizontal or vertical movements (data not shown). Total activity per hour calculated as the sum of these two parameters is presented in Figure 5.

### 3.9. Reproduction Analyses

Based on 82 litters from 45 WT dames, WT female mice produced on average 3.5 female (total 284) and 3.6 male (total 294) pups. Heterozygous OE-NPY^DBH^ female mice (35 litters from 26 dames) produced 4.0 female (total 141) and 3.8 male (total 134) pups and homozygous OE-NPY^DBH^ mice (55 litters from 34 dames) 3.3 female (total 176) and 3.7 male (total 199) pups.

## 4. Discussion

In our previous work, we observed that both the male and female transgenic mice overexpressing NPY in noradrenergic and adrenergic neurons showed increased adiposity without significant increase in body weight or food intake on a normal chow diet [6]. In the current study, we hypothesized that NPY overexpression would render the transgenic mice even more susceptible to obesity induced by a Western-type diet. In line with the hypothesis, the female OE-NPY^DBH^ mice gained significantly more weight compared to their WT littermate controls (40% versus 26% of initial weight) and showed an increased adipose tissue mass. In contrast, the male OE-NPY^DBH^ mice gained weight similarly to their WT controls (55% of initial weight). Thus, NPY overexpression overrode the resistance of the C57Bl/6 female mice [18] to the diet-induced obesity, but susceptibility of the male C57Bl/6 mice to the diet induced obesity [18] overrode the NPY's adiposity inducing effect.

The mechanisms of increased weight gain in the female OE-NPY^DBH^ mice were studied in more detail in a separate group of mice. This time the mice were homozygous for the transgene and thus housed with same-sex, same-genotype siblings, which made it possible to study the feeding behavior between the genotypes in unstressed conditions (group housing). However, the results were also verified in single-housed mice. The results showed that enhanced weight gain is not due to increased food consumption during the time preceding the difference in body weights. The mechanism seems not to be impaired thermogenesis or decreased physical activity either. Rodents resistant to the diet-induced obesity increase BAT thermogenesis via increased UCP-1 activity to avoid weight gain [19]. Similar to the OE-NPY^DBH^ and WT male mice, the female OE-NPY^DBH^ mice showed atypical BAT morphology with white fat-like appearance and large lipid vacuoles in BAT. This implied the onset of brown adipocyte degeneration often associated with obesity and impaired thermogenesis in mice [20, 21]. However, the BAT thermogenic capacity, as measured by UCP-1 expression levels in BAT, did not differ between the genotypes.

We show in this study that there are no differences in the number of litters or litter sizes between the OE-NPY^DBH^ or WT mice. Thus, prenatal and suckling conditions in this regard are similar for the pups. Therefore, different challenges in the developmental and prepubertal environment are an unlikely explanation for the weight gain in the diet-induced obesity in the OE-NPY^DBH^ mice.

Sexually dimorphic responses to NPY-associated metabolic changes have been reported in NPY Y_1_ receptor knock-out mice, which develop late-onset obesity without hyperphagia that is more pronounced in female than male mice [22]. The difference was attributed to decreased skeletal muscle mitochondrial oxidative capacity in female Y_1_ knock-out mice [23]. Gonadal steroid hormones seem to play a role in the fat accumulation in the Y_1_ knock-out mice as weight gain does not start until puberty [24, 25]. Estrogens seem to protect female mice from obesity as evidenced for instance by weight gain after gonadectomy, and their effects are at least in part mediated by hypothalamic NPY [26, 27]. Estrogens have been shown to inhibit feeding also in the brainstem [28]. Our results may imply that the overexpression of NPY in adrenergic and noradrenergic neurons overrides the protective effects of estrogens in female mice, suggesting that NPY in the brainstem and in the SNS may also play a role in mediating estrogen action on body composition.

Along with the increased adiposity, the OE-NPY^DBH^ female mice displayed impaired glucose tolerance and altered insulin sensitivity or counterregulatory effect of glucose 40 min after the ip administration of glucose. It is generally acknowledged that female mice are less likely to develop disturbances in glucose metabolism, which may be due to the antiobesity effects of estrogens in females [29, 30]. Previously, chow-fed female OE-NPY^DBH^ mice were shown to possess normal glucose tolerance despite of increased adiposity and sensitivity to stress [6, 7], and impairment in glucose tolerance in the current study occurred with increasing levels of obesity. Thus, disturbances in glucose metabolism in the female OE-NPY^DBH^ mice seem to be caused by increased adiposity rather than by direct effects of NPY or sympathoadrenal system on glucose metabolism, which is supported by a positive correlation with the area under the curve value for GTT and body weight in both female genotypes (WT, *r* = 0.54, *P* < 0.05; OE-NPY^DBH^, *r* = 0.45, *P* = 0.08). In contrast, the diet-induced hepatosteatosis that similarly affected the WT and OE-NPY^DBH^ mice is not likely to explain the impaired glucose tolerance in the female OE-NPY^DBH^ mice. However, increased glycogenolysis in the liver in response to the insulin-induced decline may be responsible for the altered insulin sensitivity presented in Figure 3(c), which remains to be studied further.

Although GTT showed very high levels of blood glucose with no genotype differences in the male mice, the OE-NPY^DBH^ mice had a tendency towards reduced insulin-induced decline in glucose levels as evidenced by the more numerous individuals that did not respond to insulin in ITT. In addition, forty per cent of the OE-NPY^DBH^ males but none of the WT littermates developed severe hyperglycaemia defined as a blood glucose level over 13.8 mmol L^−1^ following a 4-h fast. Interestingly, these tendencies occurred without major differences in WAT depot weights or at the level of hepatosteatosis in the OE-NPY^DBH^ male mice. This suggests that increased NPY may have a diabetogenic effect in the context of obesity and supports the association of the L7P polymorphism with an earlier onset of diabetes in the obese human population [14]. 

## 5. Conclusions

OE-NPY^DBH^ female mice showed a more pronounced diet-induced obesity, glucose intolerant, and insulin resistant phenotype on a Western-type energy-dense diet than their WT littermates that could not be explained by increased feeding, decreased activity, or impaired thermogenesis. This suggests that increased NPY release may predispose women and females in general, to a greater risk of weight gain under high caloric conditions. In contrast, there was no difference between the male OE-NPY^DBH^ and WT mice in the degree of obesity, although the OE-NPY^DBH^ mice seemed to be more susceptible to developing diabetes while on the diet. Interestingly, we have shown that the OE-NPY^DBH^ males fed with chow and the OE-NPY^DBH^ females with a Western diet display similar traits with the humans who carry the Proline 7 allele in their *NPY* sequence, which support the associations between the rs16139 polymorphism and high NPY levels and various metabolic risks. Furthermore, these results strengthen the hypothesis that NPY has an important role in promoting adiposity via extrahypothalamic pathways.

## Figures and Tables

**Table 1:** Metabolic and endocrinological parameters in wildtype (WT) and heterozygous OE-NPY^DBH^ mice after seven weeks on an energy-dense Western-type diet (42% kcal fat, 43% carbohydrates, 15% protein, and 0.15% supplementary cholesterol).

	Males			Females		
	WT	OE-NPY^DBH^	*P* value	WT	OE-NPY^DBH^	*P* value
	(*n* = 9)	(*n* = 10)		(*n* = 15)	(*n* = 16)	
Plasma insulin (*μ*g L^−1^)	1.4 ± 0.19	1.8 ± 0.21	0.21	0.6 ± 0.19^a^	0.5 ± 0.07^b^	0.74
Plasma cholesterol (*μ*g *μ*L^−1^)	4.3 ± 0.3	4.4 ± 0.2	0.81	1.0 ± 0.2^c^	1.3 ± 0.3^b^	0.32
Liver triglycerides (mg g^−1^ tissue)	3.0 ± 0.3	3.1 ± 0.1	0.87	4.6 ± 0.3	4.6 ± 0.3	0.96
GTT (AUC) (mmol L^−1^ × min)	1446 ± 83.0	1668 ± 113.3	0.13	961.3 ± 50.2	1115 ± 38.8	0.02

^a^ *n* = 8; ^b^ *n* = 9; ^c^ *n* = 7; ND: not determined. *P* values were obtained with a Student's *t*-test.
